# Educating novice practitioners to detect elder financial abuse: a randomised controlled trial

**DOI:** 10.1186/1472-6920-14-21

**Published:** 2014-02-01

**Authors:** Priscilla Harries, Miranda Davies, Ken Gilhooly, Mary Gilhooly, Christopher Tomlinson

**Affiliations:** 1 Brunel University, Uxbridge, UK; 2 Imperial College London, London, UK

## Abstract

**Background:**

Health and social care professionals are well positioned to identify and intervene in cases of elder financial abuse. An evidence-based educational intervention was developed to advance practitioners’ decision-making in this domain. The objective was to test the effectiveness of a decision-training educational intervention on novices’ ability to detect elder financial abuse. The research was funded by an E.S.R.C. grant reference RES-189-25-0334.

**Methods:**

A parallel-group, randomised controlled trial was conducted using a judgement analysis approach. Each participant used the World Wide Web to judge case sets at pre-test and post-test. The intervention group was provided with training after pre-test testing, whereas the control group were purely given instructions to continue with the task. 154 pre-registration health and social care practitioners were randomly allocated to intervention (n78) or control (n76). The intervention comprised of written and graphical descriptions of an expert consensus standard explaining how case information should be used to identify elder financial abuse. Participants’ ratings of certainty of abuse occurring (detection) were correlated with the experts’ ratings of the same cases at both stages of testing.

**Results:**

At pre-test, no differences were found between control and intervention on rating capacity. Comparison of mean scores for the control and intervention group at pre-test compared to immediate post-test, showed a statistically significant result. The intervention was shown to have had a positive moderate effect; at immediate post-test, the intervention group’s ratings had become more similar to those of the experts, whereas the control’s capacity did not improve. The results of this study indicate that the decision-training intervention had a positive effect on detection ability.

**Conclusions:**

This freely available, web-based decision-training aid is an effective evidence-based educational resource. Health and social care professionals can use the resource to enhance their ability to detect elder financial abuse. It has been embedded in a web resource at http://www.elderfinancialabuse.co.uk.

## Background

Financial abuse has recently been identified as the most prevalent form of elder mistreatment [1,2]. In general, rates are likely to be underestimated as studies tend to exclude those lacking cognitive capacity [1,2]; those with conditions such as dementia have been shown to be particularly vulnerable to abuse [3]. The detrimental effects of financial abuse include depression, anxiety, poverty, and deterioration in physical and mental health [4]. These effects can lead to a loss in confidence to live independently, reduced life expectancy and loss of inheritance for future generations.

Financial abuse now constitutes approximately 25% of safeguarding referrals in England [4]. The Department of Health [5], defines financial abuse as “theft, fraud, exploitation, pressure in connection with wills, property or inheritance or financial transactions, or the misuse or misappropriation of property, possessions or benefits” [p.9]. While research has shown that practitioners have some ability to identify abuse in extreme cases, they are less able to do so in more ambiguous situations [6]. They have been found to be not sufficiently alert to the warning signs of financial abuse [7]. Practitioners themselves have reported that they feel they lack the knowledge to detect elder abuse [8]. Health and social care professionals are well placed to identify financial abuse of those elders with whom they have regular contact; in this time of economic recession the need to do so has never been more urgent [9].

### Educational programmes for health and social care professionals

A range of educational programmes have been offered in the last decade to attempt to up skill the workforce; these have tended to focus on elder abuse in general rather than financial abuse specifically. In 2011, a systematic review was undertaken to examine the effectiveness of 22 such programmes, each designed to improve health and social service practitioners’ recognition and reporting of elder abuse and neglect [8]. The programmes, which ranged from short didactics to experiential learning, have generally resulted in improvements in practitioners’ awareness, collaborative activities and case identification. There was only one randomised controlled trial in the review; this examined the effect of education on knowledge and management of elder abuse [10]. The study was progressive in that it included not only outcome measures relating to informing knowledge but also impact on practice. The trial found that those that participated in a face-to-face educational course improved in ability whereas those receiving printed material did not. Interestingly the researchers found that degree of improvement was related to level of baseline knowledge; they recommended that training be tailored to level of prior knowledge. In another study, 40 trainee psychiatrists in two London National Health Service Trusts took part in a brief group educational session [11]; the intervention was found to have increased trainee psychiatrists’ knowledge and vigilance for abuse however at follow up the participants remained reluctant to ask about abuse, for fear of causing offence or harming the therapeutic relationship. As practitioners value senior colleagues opinions in practice [12] it may be that evidence based expert models are needed to give them confidence in their decisions.

There appears to be a dearth of studies that have focused on educating practitioners to identify elder financial abuse; one study has been found that reports success in promoting awareness of this “underappreciated but prevalent problem” [13]. The aim of the study was to raise awareness and assist practitioners in identifying patients at risk of elder investment fraud and financial exploitation. Initially practitioners’ views were brought together using nominal group technique to develop a set of clinically relevant screening questions, which could be used by practitioners and families to identify elder financial abuse. These were then developed and incorporated into three educational resources: a lecture, a pocket guide and a patient education brochure. These resources were provided across eight educational events in Texas to a total of 127 participants. They were well received and deemed to be of high quality. At six month follow up, it was demonstrated that of those participating in the evaluation survey, which were approximately half of the original participants, 62% had used the resources in their practice. Post training participants reported identifying a total of 25 patients, which they felt were vulnerable to elder financial abuse. It is not clear whether this is an increase as compared with pre-education levels of identification, as participants were not asked how many patients they had identified prior to taking part in the study. However the resources appear to have had a positive impact overall and the researchers advocate the value of providing more developed educational and practical tools to enhance detection [13]. This approach is also supported by the Centre for Policy on Ageing which has called for better training, particularly for health and social care professionals, to enhance professional capacity to detect financial elder abuse [14].

### Developing the evidence base for education programmes

While there have been many speculative papers about the factors thought to be associated with elder financial abuse, there has been remarkably little empirical research on how professionals actually go about making decisions in relation to the detection and reporting of elder financial abuse, or what they base their judgements upon. An evidence base, derived from the outcomes of decisions made by experienced professionals was needed.

**Figure 1:**
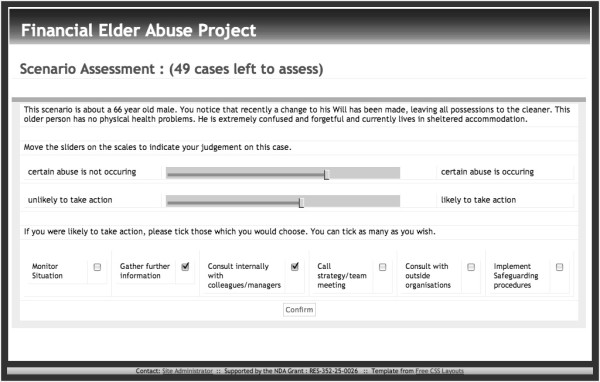
An example case scenario of elder financial abuse.

In 2008, the New Dynamics of Ageing (NDA) cross-council programme funded the authors to conduct the first in-depth study of practitioners’ decision-making with regard to financial abuse of older adults [15]. In-depth interviews with 20 health and 23 social care professionals were undertaken to identify those factors that were thought to be relevant to the decision process. Fifty computerised case scenarios were developed using these factors (see Figure 1 for an example); these were presented to 82 health and 70 social care experienced professionals.

Their decisions on the scenarios were used to statistically model how the case information had been weighted, in order to make judgements of abuse detection and likelihood of action. Two key cues were identified as influential: the mental capacity of the individual and the nature of the financial abuse occurring. It was found that 'certainty of abuse’ and 'likelihood of action’ were highly correlated (r = .98, P < .001). This implied that if novices’ ability to detect abuse could be enhanced through training, their ability to take action would also improve. The NDA study provided the first empirical evidence of how specific case information influenced experienced practitioners’ detection of elder financial abuse [16,17]. An evidence-based educational resource, to enhance novice practitioners’ ability to detect elder financial abuse, was subsequently funded for development by the E.S.R.C. in 2011 [18]. The aim of the research presented here was to test the effectiveness of that decision-training educational resource on novice health and social care professionals’ ability to detect abuse.

## Methods

### Design

A Social Judgment Theory approach was used [19]; the experts’ consensus judgements provided the criterion, against which novices’ judgements could be compared, thereby providing a measure for checking achievement. The achievement metric is the correlation between the desired judgements of the experts and the novice trainee judgements. The effect of any training can be examined by comparing the match of the novice and experts’ judgements on the cases pre- and post-test. A stronger correlation between the novice and experts’ judgements of the cases post training would indicate that the training tool was effective, as the novices’ judgements would have become more similar to those of the experienced professionals.

### Randomised controlled trial

The method was a randomised controlled trial using one intervention group and one control group; the study was conducted between January and June 2012. The use of the training information represented the independent variable; the dependent variables were the certainty of abuse ratings on the cases pre and post training. Both intervention and control groups were presented with the same case scenarios. The cases presented to the novices were also identical to those that had been presented to the expert group when the expert policies were derived [16]. The novices were asked to judge their degree of certainty that financial abuse was occurring (detection). This was done to facilitate direct comparison between expert and novices’ judgements. The certainty score ranged from 1 to 100.

The experimental condition comprised of three tasks that had to be completed consecutively: judgements on 28 scenarios; the reading of training information on how to do the task effectively and then judgements on another set of 15 scenarios (total of 43 case scenarios). The control group was asked to judge the same case scenarios, allowing them practice at making judgements about cases of financial elder abuse, but the group was not presented with the training information. No effect was therefore anticipated, as the control group had not been exposed to any training. Scenario presentation order was randomised for all participants to counter order effects. The randomised content also meant that the variation between the cases appeared subtle and each case had to be carefully scrutinised to determine how it differed.

### Intervention: the decision training aid

**Figure 2:**
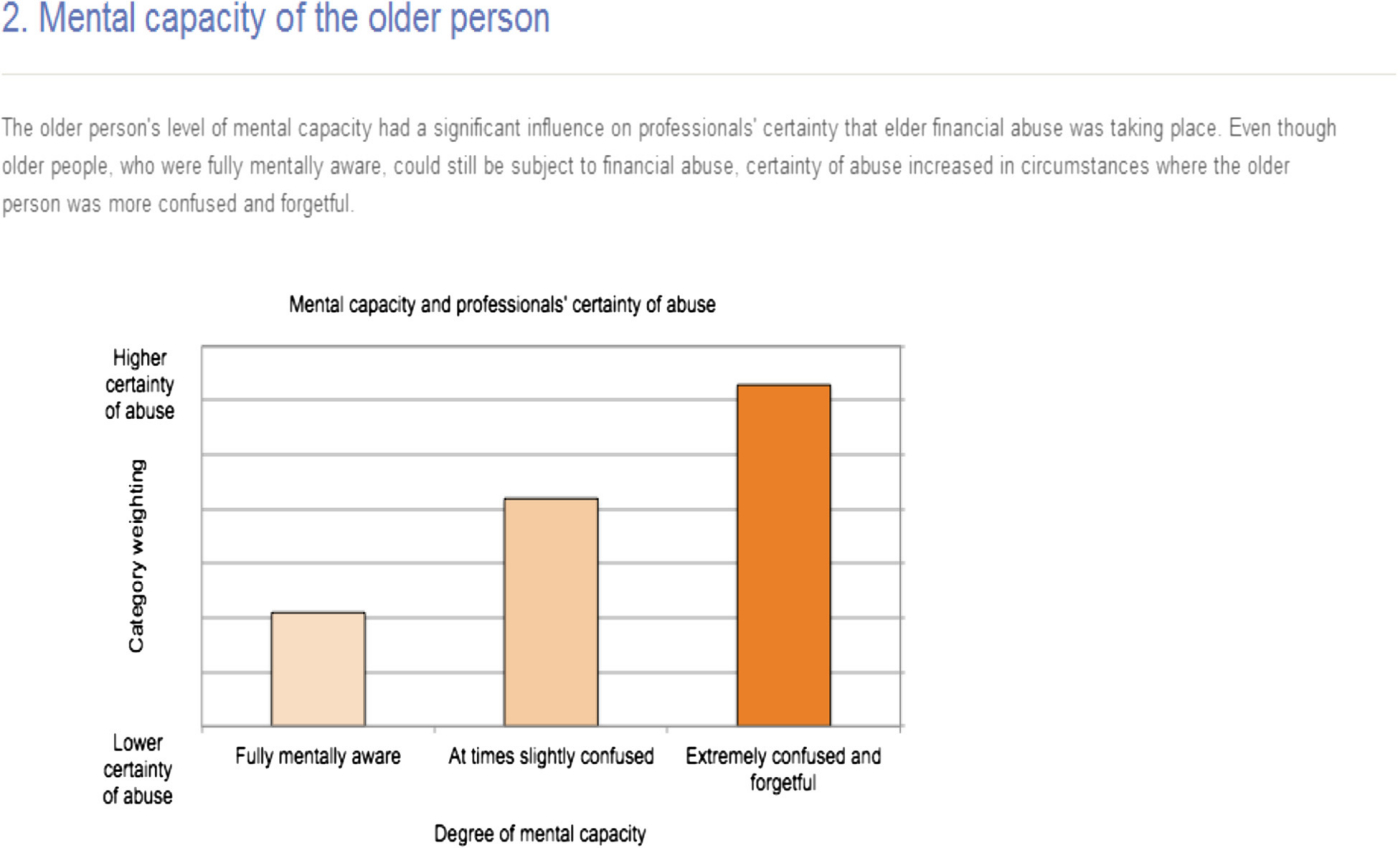
An excerpt of the training information shown to the intervention group.

The intervention informed participants how experts weighted the relative importance of the cues and the content of the cues included in the case scenarios, and as such, the sorts of factors that they should pay attention to where financial abuse is suspected [18,19]. There were seven types of cues available in the cases; these were the identifier of abuse (e.g. professional, family, friend, older person); financial problem (e.g. anomalies in finances, stealing, misuses of power of attorney, unknown befrienders and rogue traders); mental capacity and physical capacity; factors including age, gender, and living circumstances were added to contextualise the cases. The training information educated participants on how to use the cue information when making decisions; with particular regard to the most important cues needed to detect abuse, i.e. the nature of the financial problem and the client’s mental capacity [16]. Information on how to use the content of these cues was also provided (see Figure 2 as an example of a section of the training information). Graphical and descriptive information was developed and tested for ease of understanding and comprehensiveness of optimal knowledge transfer. The decision training aid test site was hosted on the World Wide Web.

### Recruitment

Permission to recruit participants was requested via 57 emails to a random selection of programme leaders of the largest pre-registration health and social care professional programmes in England (medicine, occupational therapy, physiotherapy, social work and community nursing). Those programmes, where permission was granted, were sent a recruitment email and electronic information sheet to forward to student cohorts. Students wishing to participate emailed MD for their individual password. Written consent was not required as students were seen to be giving implied consent; they had to log onto a password protected website through the World Wide Web in order to take part.

### Procedure

Participants were told they would be asked to make decisions on cases presented to them; the study was described in the information sheet as research designed to test the development of a decision-training aid.

Pre-registration students were invited to participate between January 2012 and June 2012. The recruitment invitation was sent to programme administrators to disseminate. To maintain blinding of investigators, roles were carefully organised. MD created a randomisation table in Excel (using the RANDBETWEEN (0,1) function) to allocate each individual participant to one of two conditions, either intervention, or control, and gave students their individual password if they requested to take part. KG without awareness of respondents’ allocations, undertook the analysis. Only after analysis was complete were the allocations revealed.

In the pre-test phase participants were presented with 28 cases, followed by 15 cases in the post-test phase. They were asked to indicate how certain that they were that abuse was occurring for each individual case. Although both the intervention and control group were asked to make decisions on cases of financial abuse, which could have been perceived as a form of training, between the case sets only the intervention group participants received the formal training intervention. At the same point the control group were purely told how many cases they had completed and asked to continue with the task. Participants were not told which arm of the trial they were in. Both groups undertook complete participation in all tasks in one sitting. All participants were given an honorarium on-line gift token of £10.

### Ethical approval

Brunel University School of Health Sciences and Social Care Research Ethics Committee granted ethical approval for the research. In addition some institutions also undertook approval at their own Universities ethics committees.

### Analysis

G*Power [20], an online package, was used to determine the necessary participant sample size. With a α – level of .05 and .8 power it was calculated that 48 participants would be needed in each group to identify a medium effect (*r* = .3) of the impact of the training tool on novice decision making. A medium effect size of 0.3 is described as an effect that can be clearly observed [21].

**Figure 3:**
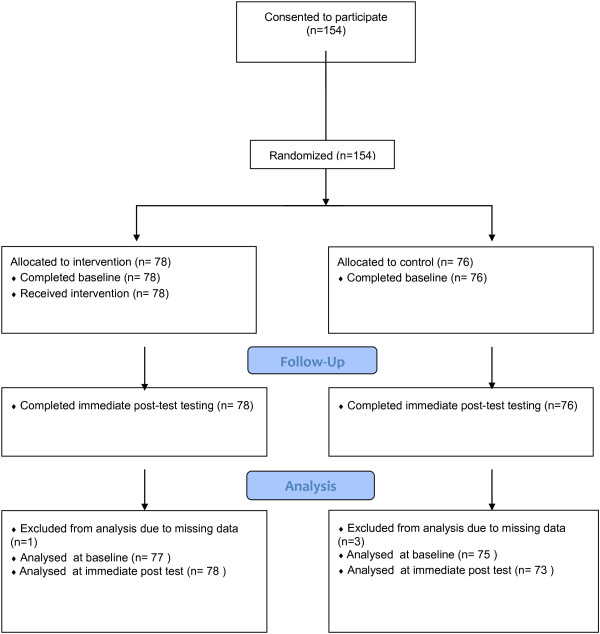
**Consort diagram [**[22]**].**

As part of the pre-analysis data checks, data was excluded where participants had left the certainty rating in the default position (scoring 50) for the majority or all of the cases; this was taken to represent missing data. One participant’s pre-test data was excluded from both from the intervention group and control group analysis and three participants’ post-test data was excluded from the control group analysis. This could have been due to their uncertainty on how to judge the cases but was more likely to have been due to apathy or a desire to complete the task quickly. Figure 3 (the CONSORT Flow Chart [22]) highlights the number of participants that were included at each stage of the analysis.

To determine the effectiveness of the training intervention, the individual participant’s rating scores were correlated with the expert scores of the same cases, using a Pearson’s correlation. Effect size was calculated by comparing the difference in the amount of change for the intervention group relative to the control group, between pre-test and post-test.

A two factor mixed design ANOVA was conducted to determine if the correlation between the experts and the intervention and control group participants was significantly different pre- and post-training. Again there was one within participants factor (Time point) having 2 levels (Pre-test and Post-test) and one between participants factor (Group) with 2 levels (Control and Intervention).

Additional analysis was conducted to compare the rating scores of the intervention and control groups, both pre and post-test. A two factor mixed design ANOVA was conducted with one within participants factor (Time point) having 2 levels (Pre-test and Post-test) and one between participants factor (Group) with 2 levels (Control and Intervention). The aim was to identify if there were any significant differences between the intervention and control group pre-test, and, to see if there was any difference post-test which could be attributed to the training intervention.

## Results

### Participants

Students (n = 154) from 11 pre-registration clinical programmes across eight universities participated. In terms of professions, 53% (n = 81) were studying medicine, 19% (n = 29) occupational therapy, 16% (n = 24) social work and 13% (n = 20) physiotherapy. In terms of stage of training, 31% (n = 47) were in the early stage, 47% (n = 72) in their mid stage and 23% (n = 35) in their final stage. They were 71% (n = 109) female, 29% (n = 45) male; 75% (n = 115) < 25 years old; 25% (n = 39) > 25 years old; 66% (n = 101) recorded their ethnicity as White, 8% (n = 12) Chinese, 6% (n = 9) Asian, 5% (n = 7) African and 16% (n = 25) of unknown ethic background.

Data was collected for 78 participants in the intervention group and 76 in the control group. Because the post-training data collection was carried out immediately following the pre-test phase, there were no participant dropouts between the two phases of data collection.

#### Effect of training on novices’ ability to detect abuse

The mean correlation of scores between the experts and the novices were identical at pre-test: the control group showing *r*_*p*_ = .45 and the intervention group also having *r*_*p*_ = .45. At post-test, the control group showed no significant change from pre-test to post-test with *r*_*p*_ = .55 (Z = .78, ns) whereas the intervention group had improved to *r*_*p*_ = .71 (Z = 2.39, p < .01) suggesting that the training tool did have an effect on novice’s capacity to detect elder financial abuse, as their ratings were more highly correlated with the experts. Table 1 shows the correlation between the scores of the experts and control and invention groups at pre- and post test.

**Table 1:** Correlation of certainty scores pre- and post-test between experts and the control and intervention groups

	**Pre-test (SD)**	**N**	**Post-test (SD)**	**N**	**Mean difference pre- to post-test (SD)**
Control	0.45 (0.22)	75	0.55 (0.23)	73	0.1 (0.28)
Intervention	0.45 (0.23)	77	0.71 (0.17)	78	0.26 (0.23)
Cohen’s d (r).					0.62 (0.3)

Effect size, calculated using the mean difference between pre- and post-test correlation scores for control (0.1, sd 0.28) and intervention (0.26, sd 0.32) demonstrated a moderate effect (d = .62, r = .3).

This result was further supported by ANOVA. This showed a significant facilitating effect of being in the Intervention Group (F (1, 148) = 5.56, p < .05, part eta sq = .04), overall a significant facilitating effect of Time point (F(1,148) = 72.75, p < .001, part eta sq = .33) and a significant interaction between Group and Time point F(1, 148) = 16.05, p < .01, part eta sq = .10), which showed a strong effect for Time point in the intervention condition but not in the control condition.

#### Effect of education on novices’ ratings of certainty of abuse

Table 2 shows means and standard deviations of certainty rating scores pre- and post-test for control group and intervention group.

**Table 2:** Certainty scores pre- and post-test for control group and intervention group

	**Group**	**Mean**	**Std. deviation**	**N**
Average certainty pre-test	Control	59.15	10.09	76
	Intervention	56.97	10.52	78
	Total	58.04	10.33	154
Average certainty post-test	Control	61.41	11.54	76
	Intervention	64.64	10.05	78
	Total	63.05	10.90	154

ANOVA showed no significant facilitating effect of Group (F (1, 152) = 0.14, ns, part eta sq = .00), a significant facilitating effect of Time point (pre versus post test) (F (1,152) = 27.07, p < .001, part eta sq = .15) and a significant interaction between Group and Time point (F(1,152) = 8.03, p < .01, part eta sq = .05). This reflects an effect for Time point in the Intervention condition but not in the Control condition. i.e. the intervention group were significantly different from the control group after receiving the training aid.

## Discussion

The results demonstrate that the educational intervention has had a positive effect on novice practitioners’ decision making; novices who received the educational intervention were more able to detect cases of financial elder abuse, than those who simply practised making decisions on cases. Through adopting the decision making approach of the experts, the novices were able to change how they used the case features to improve their decision making. In comparing the results with the one other study specifically related to elder financial abuse education [13], this finding supports the view that practitioners are able to identify cases of financial abuse post training. In the Mills et al. study [13], practitioners subjectively valued the education provided and continued use of the resources past the point of training; these types of outcome were not measured by our trial. However our study extends the findings in two ways: the effect of training is demonstrated against a control group and against pre-training levels, but most importantly the appropriate identification of cases has been confirmed as trainees’ decisions are validated against experts’ decisions on the same cases.

In comparing our results to those studies that have tested interventions to improve professionals’ ability to detect and manage elder abuse in general, not just financial abuse, our findings are consistent with those that have obtained a positive outcome of training. The advantage of our study is that the educational intervention has been developed from a very extensive and recent study of expert decision making in the field. In addition it has been designed specifically for novices; this meets the recommendation that educational interventions need to be tailored to prior levels of learning [13].

In our study, where participants read the training materials prior to making decisions about cases in the post-test phase, a positive effect was achieved. In the study by Richardson et al. [10] paper based educational materials did not have a beneficial effect, only the interactive training. Perhaps when reading educational materials of this nature, participants may not fully process the information and subsequently develop the necessary knowledge unless there is some subsequent interaction or immediate application of the educational materials. Alternatively it could be the depth and breadth of the written information presented or perhaps the expert sources from whom it has been derived that determines whether or not it has an effect on capacity.

In considering limitations of the study, our research was conducted with students rather than practitioners, meaning that the research could not measure the effect on practice; changes in practitioners’ behaviour cannot be assumed. In addition, there was no measurement of whether the effect of the training tool was maintained over time.

The study has contributed to educational research in health and social care; studies where experienced practitioners’ knowledge is captured, and good practice identified, are important in promoting effective decision making capacity relevant to real world environments. It is important that we continue to test educational approaches that can provide effective simulated decision-making environments to enhance clinical expertise [23]. The impact of enhanced ability to detect abuse can not be underestimated; health and social care professionals need to make better detection decisions, so those most at risk can be identified and protected. The detrimental effects of financial abuse and the subsequent impacts of these effects for individuals, their families and their future generations can be reduced through enhanced workforce capacity [5].

Following completion of the project in August 2012, the educational intervention together with the case scenarios, were launched as web-based decision training aid; the software for statistical analysis was added into the website so that feedback, on ability to judge abuse pre- and post-test, could be given to the user. Additional resources were also developed to accompany the case scenarios: eight podcasts were recorded with individual experts, such as the Chief Executive of Action on Elder Abuse, discussing practitioners’ responses to elder financial abuse; these were hosted alongside more detailed examples of the case scenarios to compliment the training tools. Finally seminar materials were developed and included on the website to complete the training resource repository. The website was made freely available via the World Wide Web at http://www.elderfinancialabuse.co.uk in August 2012; over 1000 individual web users were recorded in the first three months of the web resource being launched.

In Scotland the training has been used as part of the Professional Practitioner Initiative [24] and in some parts of England, practitioners have been introduced to the resource training aid county-wide [25]. To this end the education resource appears to be seen as a valuable training resource.

A plethora of reports, advice and guidance has been issued by such organisations as the Alzheimer’s Society [26], Age UK [27], and Action on Elder Abuse [28]. Comprehensive guides for practitioners have been developed to detail the legal framework, which must be adhered to in such cases [29]. Advice on how the legislation and guidance can be used to optimally support best practice has been produced [30,31]. The website, which hosts the educational training aids, compliments this range of national resources needed to support this complex area of decision making.

The evidence underpinning the educational package will need to be regularly updated to ensure it keeps pace with changes in policy and practice. For example with the full-scale introduction of Personal Independence Payments [32], in April 2013, financial abuse may alter how it presents. Prevalence of financial abuse may increase; formerly payments were managed through a system involving social care governance but with the option to receive direct payments, one mechanism for abuse detection will be removed.

## Conclusion

In summary, for older people to live happily in the community, without fear of financial exploitation, there needs to be good mechanisms in place to identify when financial abuse is occurring [33]. Health and social care professionals’ decisions can be key to such mechanisms; this educational intervention has been shown to have a positive effective on these practitioners’ ability to detect such abuse; widespread use of this training resource will help to protect elders from the detrimental effects of financial abuse.

## Abbreviations

NDA: New dynamics of ageing; CONSORT: Consolidated standards of reporting trials.

## Competing interests

The authors declare that they have no competing interests.

## Authors’ contributions

PH conceived of the study, designed the proposal, led the project team and drafted the paper. MD managed the day-to-day project, developed the data collection tools and over saw recruitment. KG performed the statistical analysis. MG advised on proposal submission and the inclusion of the expert data in the development of the training materials. CT oversaw data management and developed the web platform. All authors read and approved the final manuscript.

## Authors’ information

PH is a Reader and Divisional Director of Occupational Therapy. She has a Masters in Occupational Therapy and a PhD in Psychology. MD is a postdoctoral research fellow. She has a Masters in Health Psychology and a PhD in Health Sciences. KG is a Research Professor in Quantitative Gerontology and an expert in statistics, expertise and cognition. MG is a Professor of Health Studies and is an expert in gerontology. She was the PI of the NDA Grant [Grant no. RES-352-25-0026], which preceded this ESRC follow-on study held by PH [RES-189-25-0334]. CT is a Senior Data Systems an Integration Developer. He has an MSc in Knowledge Based Systems and a Ph.D. in Parallel Computing.

## Pre-publication history

The pre-publication history for this paper can be accessed here:

http://www.biomedcentral.com/1472-6920/14/21/prepub
